# PEEP-induced alveolar recruitment in patients with COVID-19 pneumonia: take the right time!

**DOI:** 10.1186/s13054-021-03573-x

**Published:** 2021-04-30

**Authors:** Gianmaria Cammarota, Rachele Simonte, Edoardo De Robertis

**Affiliations:** 1 Servizio di Anestesia e Rianimazione 2, Azienda Ospedaliera di Perugia, Perugia, Italy; 2 Dipartimento di Medicina E Chirurgia, Università Degli Studi di Perugia, Perugia, Italy

**To the Editor,**

Acute respiratory disease syndrome (ARDS) related to novel coronavirus-19 disease (COVID-19) is a specific pathological condition characterized, at an early stage, by normal or high respiratory system compliance and hypoxemia [1]. This so-called *“low”* phenotype of ARDS associated to COVID-19 is also characterized by a low lung weight, with ground-glass opacities located in subpleural areas at chest computed tomography scan (CT-scan), and low response to lung recruitment [1]. In case of adverse evolution of COVID-19 pneumonia and high stress ventilation as for patient-self-inflicted lung-injury induced by vigorous negative pressure developed during spontaneous breathing or non-invasively assisted breath, *“low”* phenotype may worsen in *“high”* phenotype with low respiratory system compliance, high right-to-left shunt, high lung weight, and good response to lung recruitment. Thus, while assuring a protective ventilation, a high positive end-expiratory pressure (PEEP) strategy, similar to that employed in managing severe COVID-19-free ARDS, can be pursued when a predominant *“high”* phenotype is observed [1].

In the interesting investigation by Ball and colleagues [2], the authors addressed the effects of PEEP on alveolar recruitment evaluated through CT-scan. They concluded against the adoption of high PEEP strategy because it did not lead to a substantial alveolar recruitment and worsened respiratory mechanics. However, while reading in details the investigation by Ball et all [2], some concerns raise about the modalities of PEEP application proposed. First of all, no lung recruitment maneuver was performed prior to switch from 8 to 16 cmH_2_O of PEEP. This might have led to the occurrence of lung overdistention and partial atelectasis resolution, limiting the potential positive effects exerted by PEEP in those circumstances. Indeed, in ARDS patients, lung recruitment maneuver is usually performed to normalize lung volumes both during invasive [3] and non-invasive mechanical ventilation [4]. Lastly, the assessment of lung recruitment through CT-scan was performed 1 min following the application of 16 cmH_2_O-PEEP. This time frame might not be enough to induce an improvement in the extent of aerated lung tissue and reduction of poorly/non aerated lung zones. According to Chiumello et al. [5], the evaluation of lung aeration at 5 and 15 cmH_2_O of PEEP through CT-scan and lung ultrasound was performed during a 2-h-lasting trial. Also, the changes in respiratory system compliance induced by a PEEP of 15 cmH_2_O were clinically relevant at 5 and 15 min from the baseline assessment with a 5 cmH_2_O of PEEP in intubated ARDS patients [3].

## Data Availability

Not applicable.

## Abbreviations

ARDS: Acute respiratory disease syndrome
COVID-19: Novel coronavirus-19 disease
PEEP: Positive end-expiratory pressure
CT-scan: Computed tomography scan
