# Supplementary material for: PEEP-induced alveolar recruitment in patients with COVID-19 pneumonia: take the right time!
Source: Crit Care. 2021 Apr 30;25:163. doi: 10.1186/s13054-021-03573-x (PMC8086221; doi:10.1186/s13054-021-03573-x)
Supplement: Related texts

# Reply to: PEEP-induced alveolar recruitment in patients with COVID-19 pneumonia: take the right time!

**Authors:** Lorenzo Ball, Chiara Robba, Lorenzo Maiello, Jacob Herrmann, Sarah E. Gerard, Yi Xin, Denise Battaglini, Iole Brunetti, Giuseppe Minetti, Sara Seitun, Antonio Vena, Daniele Roberto Giacobbe, Matteo Bassetti, Patricia R. M. Rocco, Maurizio Cereda, Lucio Castellan, Nicolò Patroniti, Paolo Pelosi

We thank Dr. Cammarota and colleagues for their interest in our study [2] and for giving us the opportunity to extend the discussion of our findings through this correspondence. They advocate the use of higher PEEP in patients with high-elastance COVID-19 phenotype and questioned the short time elapsed between the CT scans with PEEP at 8 and 16 cmH_2_O in our study.

The fascinating concept of using higher PEEP levels in ARDS to allow a more protective ventilation has been investigated for more than 20 years. However, despite promising initial reports, randomized trials failed to show benefits when compared to strategies aiming at maintaining oxygenation with the use of lower PEEP levels [6, 7]. Therefore, we believe that the use of higher PEEP and recruitment maneuvers should be limited to selected patients based on clinical and physiological reasoning [7], including in COVID-19 pneumonia.

Concerning the current study [2], we preferred not performing a recruitment maneuver and limiting the time spent at the CT facility for safety concerns related to the severity of COVID-19 patients. A time around 1 min of ventilation at 16 cmH_2_O of PEEP was applied before repeating the CT scan, corresponding to about 20 breaths with a plateau pressures ranging from 25 to 35 cmH_2_O. Studies showed that most changes in volume and recruitment occur in this timeframe [8] and that most respiratory units recruit below 30 cmH_2_O [9]. In fact, we were able to detect a clear recruitment effect in some patients, as illustrated in Fig. 1a, b. On the other hand, patients with low recruitment had either diffuse ground glass opacities with few non-aerated areas (Fig. 1c) or large opacities with early fibrotic progression (Fig. 1d), two conditions which might explain the lack of response to PEEP. These findings are consistent with the worsening of compliance at higher PEEP level, which we observed using a longer time-window of 5 min.

**Fig. 1:** Computed tomography at two PEEP levels in two recruiters (top panels) and two non-recruiters (bottom panels). Recruitment is computed as the difference between PEEP 8 and 16 cmH_2_O in non-aerated tissue, expressed as percentage of the total lung weight. ΔV_gas_ is the increase of lung gas volume from 8 to 16 cmH_2_O PEEP

In conclusion, our method may have underestimated the absolute magnitude of PEEP-induced recruitment in some patients; nevertheless, according to our data, the use of PEEP levels higher than those strictly required to maintain oxygenation should be avoided in severe COVID-19 pneumonia.
